# Genetic markers of white matter integrity in schizophrenia revealed by parallel ICA

**DOI:** 10.3389/fnhum.2015.00100

**Published:** 2015-03-03

**Authors:** Cota Navin Gupta, Jiayu Chen, Jingyu Liu, Eswar Damaraju, Carrie Wright, Nora I. Perrone-Bizzozero, Godfrey Pearlson, Li Luo, Andrew M. Michael, Jessica A. Turner, Vince D. Calhoun

**Affiliations:** ^1^The Mind Research NetworkAlbuquerque, NM, USA; ^2^Department of Electrical and Computer Engineering, University of New MexicoAlbuquerque, NM, USA; ^3^Department of Neurosciences, School of Medicine, University of New MexicoAlbuquerque, NM, USA; ^4^Departments of Psychiatry, Yale University School of MedicineNew Haven, CT, USA; ^5^Olin Neuropsychiatry Research Center, Institute of LivingHartford, CT, USA; ^6^Department of Internal Medicine, University of New MexicoAlbuquerque, NM, USA; ^7^Department of Psychology and Neuroscience Institute, Georgia State UniversityAtlanta, GA, USA

**Keywords:** diffusion tension imaging (DTI), fractional anisotropy (FA), parallel independent component analysis (P-ICA), single nucleotide polymorphisms (SNPs), schizophrenia

## Abstract

It is becoming a consensus that white matter integrity is compromised in schizophrenia (SZ), however the underlying genetics remains elusive. Evidence suggests a polygenic basis of the disorder, which involves various genetic variants with modest individual effect sizes. In this work, we used a multivariate approach, parallel independent component analysis (P-ICA), to explore the genetic underpinnings of white matter abnormalities in SZ. A pre-filtering step was first applied to locate 6527 single nucleotide polymorphisms (SNPs) discriminating patients from controls with a nominal uncorrected *p*-value of 0.01. These potential susceptibility loci were then investigated for associations with fractional anisotropy (FA) images in a cohort consisting of 73 SZ patients and 87 healthy controls (HC). A significant correlation (*r* = −0.37, *p* = 1.25 × 10^−6^) was identified between one genetic factor and one FA component after controlling for scanning site, ethnicity, age, and sex. The identified FA-SNP association remained stable in a 10-fold validation. A 5000-run permutation test yielded a *p*-value of 2.00 × 10^−4^. The FA component reflected decreased white matter integrity in the forceps major for SZ patients. The SNP component was overrepresented in genes whose products are involved in corpus callosum morphology (e.g., *CNTNAP2, NPAS3*, and *NFIB*) as well as canonical pathways of synaptic long term depression and protein kinase A signaling. Taken together, our finding delineates a part of genetic architecture underlying SZ-related FA reduction, emphasizing the important role of genetic variants involved in neural development.

## Introduction

Schizophrenia (SZ) is a severe, debilitating mental disorder with heritability estimated up to ~80% (Sullivan et al., 2003). However, its genetic basis remains elusive. The last decade has seen a large number of genome wide association studies (GWAS) searching for genetic variants regulating the liability for SZ (Gejman et al., 2011). More recently, very large sample sizes have been made possible with aggregated data through collaborative consortia, yielding more reliable and replicable findings (Visscher et al., 2012). Indeed, evidence accumulates to suggest the polygenicity of SZ, where genetic variants with subtle effects may function together to confer the liability (Purcell et al., 2009, 2014; Giusti-Rodriguez and Sullivan, 2013). One estimation is that, together, genome-wide SNPs capture 23% of the variation in SZ liability, a substantial portion of which is due to common variants (Lee et al., 2012). Obviously, the combined effect of multiple variants is more prominent than those of individuals SNPs and hence more likely to be identifiable. In this sense, multivariate approaches assessing multiple variables for aggregate effects appear to be better positioned for modeling the polygenic basis of SZ.

Given the complexity and heterogeneity of SZ, one valuable strategy to unravel the genetic risk is through investigating the effects of genetic variants on intermediate phenotypes such as disrupted brain structure and function, which are more proximal to biological mechanisms compared to behavioral measures (Rose and Donohoe, 2013). In the last two decades notable progress has been made in mapping gray matter abnormalities in SZ. The investigation of white matter integrity was lagging behind until the advancement in magnetic resonance diffusion imaging (MR-DI) technique affords characterizing fiber tract direction and organization. One can model these data with a single tensor model in which a diffusion tensor is estimated for water molecules at each voxel to derive metrics of anisotropy which infer fiber properties and can be compared across subjects for associations with other traits of interest. With this technique and more complex multiple tensor or diffusion spectrum imaging techniques applied to SZ studies, there is consensus that white matter integrity is compromised in affected patients, including decreased fractional anisotropy (FA) in prefrontal and temporal lobes, as well as the fiber bundles connecting these regions (Kubicki et al., 2007). In addition, family studies further showed a genetic factors might underlie the FA deficits, which were also observed, with a lower extent, in unaffected relatives of patients (Hoptman et al., 2008; Maniega et al., 2008; Clark et al., 2011).

These previous findings strongly indicate FA deficits as a heritable biomarker for SZ. However, to our knowledge, there has not been enough work characterizing the genetic aspects, and this is what motivated the current study. We studied 73 SZ patients and 87 controls with good-quality genome-wide SNP data and FA images. A pre-filtering step was applied to locate SNPs which show a weak differentiation of patients from controls. These potential causal loci were then analyzed for associations with FA images using parallel independent component analysis (P-ICA) (Liu et al., 2009; Liu and Calhoun, 2014). As a multivariate approach, P-ICA extracts genetic and imaging components, respectively from the SNP data and FA images to capture clusters of SNPs or voxels exhibiting co-variations across subjects which simultaneously emphasizing imaging-genetic associations in order to identify genetic factors explaining FA variation. This approach also enables us to study genetic variants clustered into components at a pathway level, potentially providing more insight into the underpinnings of SZ. To guard against false discoveries, the identified FA-SNP associations were evaluated with 10-fold validation and permutation tests.

## Materials and methods

### Participants

The Mind Clinical Imaging Consortium (MCIC), a collaborative effort of four research teams from New Mexico (NM), Boston (MGH), Iowa (IOWA), and Minnesota (MINN) was involved in subject recruitment and data collection (Gollub et al., 2013). The number of healthy controls/patients with schizophrenia were balanced and matched for age/gender as much as possible. Only participants having good quality DTI and GWS data were considered. Each site's institutional review board approved the study and all the participants provided written informed consents. Healthy participants were screened to ensure that they were free of any neurological, medical or psychiatric illnesses, including any history of substance abuse. Structured clinical interviews for DSM-IV (SCID) and case file reviews confirmed a diagnosis of schizophrenia for the patients (Williams et al., 1992). A total of 160 participants who had volunteered for DTI and gene testing were included in this work, including 73 SZ patients and 87 healthy controls (see Table 1 for demographic information). We admitted all ethnic groups (self-reported) to maximize the sample size. Ethnicity information was missing for eight participants but was inferred based on the genetic data using principal component analysis (PCA).

**Table 1:** **Demographic information of patients with schizophrenia and healthy controls**.

**Demographics**		**SZ (73)**	**HC (87)**
Sex	Male	54	51
	Female	19	36
Age	Mean ± SD	34 ± 11	32 ± 11
Ethnicity	Caucasian	53	81
	Asian	5	3
	American Indian or Alaska Native	1	0
	African-American	14	3

### DTI image acquisition and preprocessing

The image acquisition parameters are summarized in Table 2 (White et al., 2011). Prior to diffusion tensor calculation, images went through standard preprocessing (Caprihan et al., 2011). DTI preprocessing included three steps: (1) *Data quality check:* Subjects with signal dropouts caused by motion or presence of striated artifacts on images were not included in the study. (2) *Motion and eddy current correction:* We registered all the images to a *b* =0 sec/mm^2^ image. Twelve degrees of freedom, affine transformation with mutual information cost function was used for image registration. (3) *Adjusting the diffusion gradient direction:* Two corrections were applied to the diffusion gradients. The nominal diffusion gradient directions were prescribed in the magnet axis frame. We rotated them to correspond to the image slice orientation. No correction was required if the imaging slice was pure axial. A second correction accounted for any image rotation during the previous motion and eddy current correction step. The rotation part of the transformation found previously was extracted, and each gradient direction vector corrected accordingly. All the image registration and transformations were done with the FLIRT (FMRIB's Linear Image Registration Tool) program [FMRIB Software Library (FSL); www.fmrib.ox.ac.uk/fsl]. Dtifit, a tool in FSL, was adopted to calculate the diffusion tensor and the fractional anisotropy (FA) maps. The FA image was aligned to a MNI FA template with a non-linear registration algorithm FNIRT (FMRIB's Non-linear Image Registration Tool; FSL) and resliced via SPM resulting in a final voxel size of 2 × 2 × 2 mm. After preprocessing, the FA maps were smoothed using 8 mm smoothing kernel (Jones et al., 2005).

**Table 2:** **DTI acquisition parameters at four sites for MCIC study**.

**Scanning parameter**	**Site 1**	**Site 2**	**Site 3**	**Site 4**
Scanner (Tesla)	Siemens (1.5)	GE Signa (1.5)	Siemens Trio (3)	Siemens (1.5)
TR (ms)	9500	8900	10,500	9800
TE (ms)	90	80	98	86
Voxel dimensions (mm)	2 × 2 × 2	2 ×2 × 2	2 × 2 × 2	2 × 2 × 2
Diffusion directions	6	60	12	12
*B*-values (s/mm^2^)	0/1000	0/700	0/1000	0/1000
NEX	4	1	2	4
Bandwidth (Hz/pixel)	1954	1860	1342	1502

*TR, repetition time; TE, echo time; NEX, number of excitations*.

We noticed significant differences in data collected at one site, which might be related to a cohort effect or greater variability due to fewer DTI directions. With site coded as dummy variable, we performed a voxelwise regression on FA images using Matlab, thereby eliminating the site effects. Site-by-diagnosis interactions were checked *post-hoc* after P-ICA decomposition using the FA loadings.

### SNP data collection and preprocessing

The genotyping and genetic quality control procedures were same as described in our previous work (Chen et al., 2012b), which is briefly discussed here. DNA was extracted from a blood sample collected at individual sites. Genotyping for all subjects was performed at the Mind Research Network using the Illumina Infinium Human Omni1-Quad assay spanning a total of 1,140,419 SNP loci. BeadStudio was used to make the final genotype calls and PLINK (Purcell et al., 2007) was employed for a series of quality controls (Anderson et al., 2010) including: (a) gender consistency check, (b) sample relatedness (not closer than second degree relatives), (c) genotyping call rate (>90% at both the individual and SNP level), (d) Hardy–Weinberg equilibrium in the control population (*p* < 1 × 10^−6^), (e) minor allele frequency (MAF > 0.05), and (f) missing calls were replaced using high linkage disequilibrium (LD) loci if available or otherwise removed. A total of 777,365 SNP loci were retained after quality control and discrete numbers were then assigned to the categorical genotypes: 0 (no minor allele), 1 (one minor allele), and 2 (two minor alleles). With PCA, two principal components were identified as ethnicity-related and eliminated from the data. No clear population structure was observed in the corrected data.

### Algorithm for extraction of linkages in multimodal datasets

P-ICA first extracts components from the two modalities in parallel based on infomax ICA (Bell and Sejnowski, 1995), and then enhances the correlations between pairs of components loadings. The following Equations (1) and (2) describe the mathematical model of Infomax. For each modality, the data are decomposed into a linear combination of underlying components. *X*_1_ and *X*_2_ represents the FA and SNP modalities; *S*_1_ and *S*_2_ represents the underlying independent components, while *A*_1_ and *A*_2_ represent the mixing matrix/loadings for FA and SNP modalities; *W* denotes the un-mixing matrix, which is the (pseudo) inverse of *A*. Equation (3) illustrates the objective function of entropy and correlations computed between columns of loadings matrices *A*_1_ and *A*_2_. *f_y_*(*Y*) is the probability density function of *Y*; *E* is the expected value; *H* is the entropy function; *W*_0_ is the bias vector. *W* is then iteratively updated based on the natural gradient rule to optimize the objective function. A full description as well as the mathematical/simulation details of the algorithm can be found in our previous publications (Liu et al., 2008, 2009; Chen et al., 2012a) and the algorithm is available in the fusion ICA toolbox (FIT) (http://mialab.mrn.org/software/fit).

(1) $$X_{1}=A_{1}\cdot S_{1}\overset{W_{1=A_{1}^{-1}}}{\to }\;S_{1}=W_{1}\cdot X_{1}$$

(2) $$X_{2}=A_{2}\cdot S_{2}\overset{W_{2=A_{2}^{-1}}}{\to }S_{2}=W_{2}\cdot X_{2}$$

$$\begin{array}{l} \text{max}\{H\left(Y_{1}\right)+\;H\left(Y_{2}\right)+{\left[\text{corr}\left(A_{1},\;A_{2}\right)\right]}^{2}\}=\{\text{​}-E\left[lnf_{y}\left(Y_{1}\right)\right]-\\ \;E\left[lnf_{y}\left(Y_{2}\right)\right]+\;\frac{cov{\left(A_{1i},\;A_{2j}\right)}^{2}}{var\left(A_{1i}\right)\;\cdot \;var\left(A_{2j}\right)}\} \end{array}$$

(3) $$Y=\frac{1}{1+e^{-U}};\;U=WX+W_{0}$$

P-ICA yields best performances when the ratio of sample size to the number of variables is above 0.02 for the genetic modality (Liu et al., 2008). Consequently, a pre-filtering step was conducted to locate 6527 SNPs, discriminating patients from controls with *p*-values less than 0.01 uncorrected. These potential susceptibility loci were then analyzed for associations with neurobiological traits. The component number was estimated to be nine for the FA modality using minimum description length (MDL) (Rissanen, 1978). The SNP component number was estimated to be seven based on component consistency as in Chen et al. (2012a). P-ICA was performed through the Fusion ICA Toolbox (FIT, http://mialab.mrn.org/software/fit/index.html). The algorithm was configured with a threshold of 0.3 for constrained correlations to avoid false positive associations and to only constrain one pair of components. The endurance parameter was set to −1 × 10^−5^ to control the decreasing slope of the entropy term and avoid over fitting. We performed two independent P-ICA decompositions, one with the entire dataset (i.e., 73 Sz and 87 HC) and the second using Caucasian participants only (i.e., 53 Sz and 81 HC). We also performed association analysis between the FA/SNP loadings and the Positive and Negative Syndrome Scale (PANSS) symptoms as well as medication.

### Permutation and 10-fold cross validation tests

We performed a permutation test to assess the validity of identified FA-SNP association by investigating the occurrences of inter-modality correlations by chance in permuted FA and SNP datasets. The null distribution was constructed with the top correlation obtained from each test run. We then counted the instances with correlations greater than that observed from the original data and calculated the tail probability as the significance level. We also performed a 10-fold cross-validation to examine the reproducibility of the identified FA-SNP association using a subset of 90% subjects. The same P-ICA parameters were used for all subset P-ICA decompositions.

## Results

P-ICA identified one FA-SNP pair components presenting a significant correlation (*r* = −0.37, *p* = 1.25 × 10^−6^) and passing the Bonferroni threshold of 7.93 × 10^−4^, after controlling for age/sex and correcting for 63 independent tests. The FA-SNP pair replicated in all 10-fold validations having a correlation range of 0.18–0.46 and a median of 0.33. The FA and SNP components identified in subset decompositions in 10-fold cross-validation showed similar patterns to those identified with the entire dataset. The overlapping ratio refers to the percentage replication of the associated SNP component from the entire dataset with the associated SNP component in various subset decompositions. The overlapping ratio was around 50–70% in the various subset decompositions. Though variability existed in SNP component from entire dataset and subset decompositions, the genes/pathways obtained were very similar. Hence the final set of associated SNPs was selected from the results of entire dataset. In a 5000-run permutation test, the absolute values of FA-SNP top correlations ranged from 0.11 to 0.43 with a median of 0.19, yielding a *p*-value of 2.00 × 10^−4^ and passing the Bonferroni threshold of 7.93 × 10^−4^ for the identified association. We further examined the FA-SNP association within the SZ and HC groups, respectively, and observed a partial FA-SNP correlation of −0.1304 (*p* = 0.27) within the schizophrenia patients and −0.0019 (*p* = 0.98) within the healthy controls.

**Figure 1:**
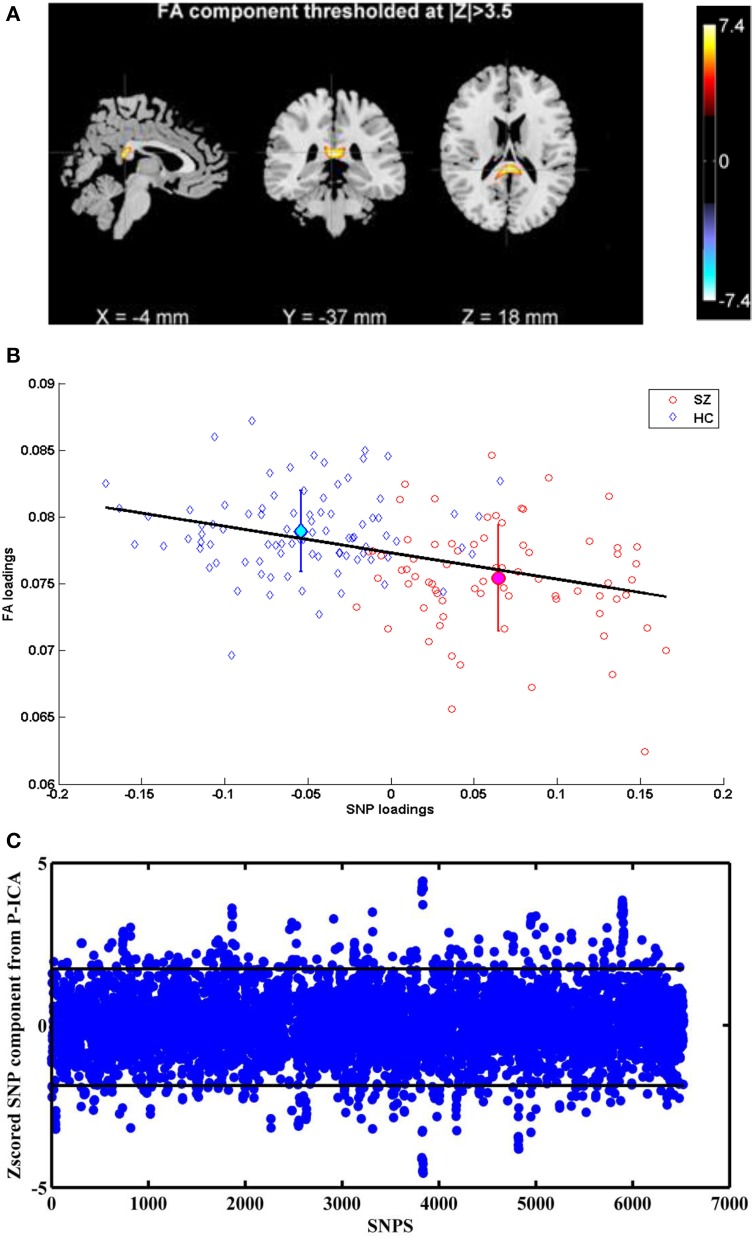
**(A)** FA component corresponding to Top P-ICA pair. **(B)** Scatter plots of FA and SNP loadings (both SZ and HC participants) from P-ICA with the regression line. The group mean directionality being HC > SZ. **(C)** Z-scored SNP component corresponding to Top P-ICA pair. Horizontal lines indicate the threshold to obtain the top 5% SNPS.

Figure 1A illustrates the spatial map of highlighted brain regions when the linked FA component was thresholded at |*Z*| > 3.5, covering 55% of the forceps major tract. The group mean of FA loadings was significantly lower in the patients with schizophrenia (*p* = 8.51 × 10^−9^), as shown in Figure 1B, reflecting decreased FA in SZ patients in the forceps major. The same direction of group mean for FA loadings (i.e., healthy controls > patients with schizophrenia) was observed at all four sites, thereby indicating no site by diagnosis interaction. We did not observe any significant FA associations with patient symptoms or chlorpromazine equivalent medication scores calculated from all current medications, which is presented in Table 3. A recent diffusion MRI study in schizophrenia reported an association between visual hallucinations and the microstructure of forceps major (Amad et al., 2013). Our dataset included 20 participants (i.e., within 73 Sz) who had a Global Rating of Severity of Hallucinations (GR_Hallu) greater than three (Range being 0–5; with five indicating most severe). On trying to replicate this however, we could not find any correlations between GR_Hallu and the FA loadings in this complementary analysis, which could be due to the very small sample size.

**Table 3:** **Summary of clinical information**.

**Number of Sz**	**% Reporting duration of illness (DOI)**	**DOI Mean ± SD**	**% Reporting (PANSS positive)**	**PANSS positive Mean ± SD**	**% Reporting (PANSS negative)**	**PANSS negative Mean ± SD**	**% Reporting medication**	**Cpz eqvt of all current medications (mg/day)**
73	95.89	11.18 ± 10.5	97.26	15.94 ± 4.9	97.26	16.06 ± 4.9	91.78%	653.52 ± 684.14

The linked SNP component depicted in Figure 1C also showed significant group difference (*p* = 1.62 × 10^−32^) as expected, since the SNPs for P-ICA were selected based on group difference. As in Figure 1B, patients presented positive loadings while controls presented negative loadings. This component was predominantly contributed to by 326 SNPs (top 5% based on absolute values of component weights). These SNPs, their host genes, z-scores of component weight, original genotypic group difference and minor allele frequencies (MAFs) in patient and control groups are indicated in Table S1. One hundred and sixty three SNPs were mapped to 90 unique genes using SNPnexus (www.snp-nexus.org), while the rest were from inter-genic regions. We also examined the genetic architecture of our finding using Ingenuity Pathway Analysis (IPA: https://www.ingenuity.com) where the 90 genes were compared with the whole genome as background. IPA identified enrichment in abnormal morphology of corpus callosum with gene hits in *CNTNAP2, NFIB*, and *NPAS3* (*p* = 2.78 × 10^−4^), as illustrated in Table 4A. IPA also revealed a number of enriched canonical pathways, including synaptic long term depression (LTD) (*p* = 8.71 × 10^−4^) and protein kinase A (PKA) signaling (*p* = 1.02 × 10^−3^), as shown in Table 4B.

**Table 4:** **Ingenuity pathway analysis on the SNP component**.

	***p*-Value**	**Molecules**
**A. DISEASES OR FUNCTIONS ANNOTATION**		
Abnormal morphology of brain	6.96E-03	CNTNAP2, FA2H, GRID2, LRRTM4, NPAS3, NFIB
Abnormal morphology of corpus callosum	2.78E-04	CNTNAP2, NPAS3, NFIB
Abnormal morphology of hippocampus	5.11E-03	LRRTM4, NPAS3, NFIB
Morphology of brain cells	6.59E-03	FA2H, GRID2, NFIB
**B. CANONICAL PATHWAYS**		
Synaptic long term depression	8.71E-04	GRID2, RYR3, RYR2, PRKCB
Protein kinase A signaling	1.02E-03	PTPRD, RYR3, RYR2, CREB5, PTPRM, PRKCB
Gαs signaling	4.79E-03	RYR3, RYR2, CREB5
Hepatic cholestasis	8.71E-03	SLCO3A1, FABP6, PRKCB
CREB signaling in neurons	1.58E-02	GRID2, CREB5, PRKCB
Calcium signaling	1.74E-02	RYR3, RYR2, CREB5

We also analyzed the FA-SNP pair obtained from the independent secondary P-ICA decomposition (i.e., considering caucasians participants), observing the FA component covered the same region of forcep majors as obtained with the entire dataset. The overlapping ratio of snps between SNP components (i.e., between caucasians only and the entire dataset P-ICA decompositions) was around 30%. Our previous studies have shown that this overlap with such small sample size is much higher than random (Chen et al., 2012a,b). We also observed a high correlation of -0.35 between the top paired FA and SNP loadings in this caucasians only decomposition.

## Discussion

In this study we investigated the genetic underpinnings of white matter abnormalities in SZ. A multivariate approach, P-ICA, was used to extract SNP and FA components, and retrieve inter-modality associations. Due to the limited sample size compared to genome-wide SNPs, we preselected 6527 risk loci based upon group difference to focus the FA-SNP association analysis on polymorphisms likely relevant to SZ. This strategy could bias the significance level of group difference in the SNP component, but not the significance of identified FA-SNP correlations. Finally one significant FA-SNP pair was identified with its validity confirmed by the permutation test and 10-fold cross validation.

The identified FA component highlighted forceps major with the loadings indicating lower FA-values in SZ than HC. Forceps major fibers are located in the posterior end of the corpus callosum (CC), also known as splenium. These fibers interconnect bilateral parietal and occipital (dorsal portion), as well as temporal cortices (ventral portion) (Park et al., 2008). There is a growing body of evidence that FA is decreased in splenium for SZ patients (Cheung et al., 2008; Gasparotti et al., 2009; Knoche et al., 2012; Ellison-Wright et al., 2014), which has also been confirmed in meta-analyses (Ellison-Wright and Bullmore, 2009; Patel et al., 2011). Splenium was also identified to show decreased FA in an ICA based study (Caprihan et al., 2011). Note that this local FA reduction is observed in both chronic and non-medicated first-episode patients, indicating a low possibility of medication effect, consistent with no association being observed between the FA loadings and the medication dosages in our study. Instead, the lower FA may be a consequence of demyelination or axon loss (Alexander et al., 2007), which coincides with the reports of SZ-related reduction in volume of CC and the subregion of splenium (Arnone et al., 2008; Francis et al., 2011). Additionally, family and twin studies suggest the FA reduction in splenium and alterations in CC structure are likely regulated by genetics (Pfefferbaum et al., 2001; Francis et al., 2011; Knoche et al., 2012). Overall, the identified imaging component revealed a white matter biomarker robustly identified to characterize SZ while also exhibiting heritability.

A negative FA-SNP correlation was observed; reflecting participants with SZ carrying higher loadings of specific genotypes had lower FA-values in forceps major fibers. Corresponding to the FA findings in splenium, the SNP component was overrepresented in networks affecting the abnormal morphology of corpus callosum, involving *CNTNAP2, NPAS3*, and *NFIB* genes, as in Table 4A. *CNTNAP2* encodes a neurexin protein involved in neuron interaction and axon differentiation. SNPs in *CNTNAP2* have shown associations with FA in uncinated fasciculus (rs2710126) (Clemm von Hohenberg et al., 2013) as well as graph measures of structural brain connectivity including small-worldness and global efficiency (rs2710102) (Dennis et al., 2011). A copy number variation disrupting *CNTNAP2* is also suggested to play a role in SZ (Friedman et al., 2008). The *NPAS3*-encoded protein regulates neurodevelopment (Sha et al., 2012) and NPAS3-deficient mice exhibited hypoplasia of CC (Brunskill et al., 2005). Common SNPs in *NPAS3* have also been associated with SZ (Macintyre et al., 2010). The transcription factor *NFIB* is demonstrated to play a central role in the maturation of midline glia and formation of CC in mouse studies (Piper et al., 2009). One speculation based on our finding is that these identified SNPs affect white matter integrity through regulating brain development, and SZ patients tend to carry more risk genotypes for low white matter integrity.

The SNP component was also enriched in PKA signaling, involving *CREB, PRKCB, RYR2, RYR3, PTPRD*, and *PTPRM*, as in Table 4B. Cyclic AMP-dependent protein kinase (PKA) is an enzyme responsible for the phosphorylation of numerous nervous system proteins (Daniel et al., 1998), thus it is involved in various mechanisms including axon guidance and myelination (Yoon et al., 2008; Murray et al., 2009). For instance, PKA promotes the phosphorylation of CREB which regulates the transcription of BDNF, a protein documented in neurogenesis, axon guidance and synaptic plasticity (Cohen-Cory et al., 2010). Indeed, CREB has a pivotal role in cellular processes and is implicated in a wide spectrum of disorders including SZ (Yuan et al., 2010; Saura and Valero, 2011). *PRKCB* encodes calcium-dependent protein kinase (PKC) beta whose activity is shown to modulate myelin gene expression in enriched oligodendrocytes (Asotra and Macklin, 1993) and mediate axon regeneration (Sivasankaran et al., 2004). PKC beta is also observed to exhibit disrupted expression in chronic SZ (Hakak et al., 2001). *RYR2* and *RYR3* encode ryanodine receptors serving as a calcium release channel, thus they are involved in synaptic plasticity (Caillard et al., 2000; Adasme et al., 2011) and functions in axon degeneration through mediating operations of GluR4 AMPA receptors (Ouardouz et al., 2009). *PTPRD* and *PTPRM* encode receptor protein tyrosine phosphatases delta and mu which are significantly involved in neural development and axon guidance (Ensslen-Craig and Brady-Kalnay, 2004). It is interesting to note that, again the highlighted genes appear to converge on their developmental roles.

Another significantly enriched pathway, LTD, involved *GRID2* and three genes also participating in PKA signaling (RYR2, RYR3, and PRKCB). GRID2 is a glutamate receptor known for its role in synapse formation and LTD induction in cerebellum (Yuzaki, 2013). How it may relate to white matter integrity awaits further investigations. The other three genes, as reviewed above, have been implicated in both axonogenesis and synaptic plasticity, which is also documented for other genes (*CREB, PTPRD*, and *PTPRM*) highlighted in the SNP component. While the SZ neuropathology hypothesis based on altered brain connectivity diverges in whether the dysconnection manifests anatomically (through structural changes of association fibers) or functionally (through aberrant control of synaptic plasticity), our observation suggested that these two factors might coexist (Stephan et al., 2009). Despite that the FA-SNP association indicated these genes contributing to structural dysconnectivity, their potential role in abnormal synaptic regulation cannot be ruled out and deserves further delineation.

The current study was limited in the following aspects. First, the imaging data were collected at multiple sites with different platforms. To address the potentially confounding site effect, we performed a voxelwise regression to eliminate the related effects and used the corrected data for P-ICA. Another limitation lied in the admission of subjects with various ethnicities. To avoid biased findings, we corrected the SNP data by eliminating the ethnicity-related components. In the resulting FA-SNP association, we did not observe any significant contribution from ethnicity. Meanwhile, due to the limited sample size, the investigation of FA-SNP associations focused on 6527 pre-filtered SNPs, which is likely incomplete given the complex mechanisms underlying white matter abnormalities. Our finding depicts a part of the picture. The observed SNP-FA association appeared to be mainly driven by the group difference, given that no significant correlation was observed in either patient or control group. Specifically for the patient group, we included medication (i.e., chlorpromazine equivalents of current medication) in the regression model and did not observe any significant effect on the FA loadings. Exploring further we found that the top paired SNP component from P-ICA was found to be the second most significant component showing group difference, replicating reliably in all subset evaluations. This could indicate the uniqueness in FA-SNP component association other than the shared group difference, bringing forth the fact that FA changes are likely due to genetic factors. However, other factors cannot be completely ruled out in this work. Though premature, we might speculate that the FA-SNP relationship is limited by the range of symptom severity measures. The range for PANSS positive and negative symptoms is 0–49 (Opler et al., 2006) and from Table 3 we observe that this dataset could represent a population occupying a very narrow slice of the schizophrenia spectrum. The association therefore, might get stronger in a dataset that is more strongly psychotic. Also, the unavailability of an independent dataset for replication of results is a limitation, though we ascertained this to a certain extent using 10-fold validation and permutation tests. FA-SNP association presented here is the first step and future work will involve investigation of complementary indices like axial diffusivity and radial diffusivity, for association with genetic data. We chose the FA approach as it enables a full characterization of multivariate distribution of values. Future work may also focus on the use of Tract Based Spatial Statistics, which provides a much smaller set of values and removes data in a complex and non-linear manner.

In conclusion, we consider this work one step further toward understanding the genetic underpinnings of white matter abnormalities in SZ, in part as it assessed multiple genetic variants for aggregate effects. More importantly, we revealed that this local FA reduction was associated with a genetic factor enriched in CC morphology, LTD and PKA signaling. The involved genes appear to converge on developmental functions, indicating a possibility that genetic variants contribute to white matter abnormalities through disrupting neural development. The fact that these genes also participate in synaptic plasticity leads to the speculation of functional dysconnectivity, which will be investigated in a future study.

### Conflict of interest statement

The authors declare that the research was conducted in the absence of any commercial or financial relationships that could be construed as a potential conflict of interest.
