# Supplementary material for: Genetic markers of white matter integrity in schizophrenia revealed by parallel ICA
Source: Front Hum Neurosci. 2015 Mar 3;9:100. doi: 10.3389/fnhum.2015.00100 (PMC4347454; doi:10.3389/fnhum.2015.00100)
Supplement: Supplementary file 1 [file Table1.PDF]

Supplementary Material-Table S1: Top 5% SNPs identified from the SNP component

Snps	Gene	Chromosome	PICAWeights	MAF(Sz)	MAF(HC)	P value
rs7904014	PTER	chr10	-4.557	0.472	0.540	0.227
rs10904747	PTER	chr10	-4.500	0.5	0.5	1
rs10795400	PTER	chr10	-4.448	0.376	0.448	0.188
rs7904568	PTER	chr10	4.436	0.472	0.454	0.744
rs7092037	PTER	chr10	4.419	0.410	0.431	0.724
rs1411877	PTER	chr10	4.304	0.458	0.534	0.174
rs10466288	PTER	chr10	-4.270	0.431	0.436	0.924
rs10904737	PTER	chr10	4.228	0.417	0.425	0.895
rs10904757	PTER	chr10	4.207	0.458	0.534	0.201
rs12572781	PTER	chr10	4.187	0.445	0.459	0.801
rs10904748	PTER	chr10	-4.149	0.513	0.488	0.647
rs7922050	PTER	chr10	-4.128	0.410	0.431	0.724
rs4028374	-	chr10	-4.115	0.410	0.482	0.180
rs10904730	-	chr10	4.110	0.5	0.488	0.832
rs7093030	PTER	chr10	-4.082	0.410	0.402	0.878
rs2644267	FHOD3	chr18	3.847	0.390	0.471	0.136
rs9528946	-	chr13	-3.812	0.390	0.471	0.136
rs9528947	-	chr13	-3.812	0.315	0.178	0.005
rs591873	FHOD3	chr18	3.750	0.349	0.454	0.047
rs2957176	FHOD3	chr18	3.741	0.321	0.247	0.135
rs754040	FHOD3	chr18	3.728	0.301	0.160	0.004
rs7905643	PTER	chr10	3.710	0.397	0.298	0.054
rs7991583		chr13	-3.695	0.458	0.459	0.987
rs527797	FHOD3	chr18	3.676	0.349	0.252	0.052
rs2644262	FHOD3	chr18	3.638	0.328	0.195	0.007
rs6829892		chr4	3.603	0.404	0.321	0.103
rs4603639	FHOD3	chr18	3.567	0.452	0.471	0.732
rs2644266	FHOD3	chr18	3.547	0.383	0.293	0.071
rs9540481		chr13	-3.540	0.301	0.183	0.021
rs879567	FHOD3	chr18	3.521	0.445	0.293	0.002
rs10216540		chr8	3.482	0.397	0.454	0.294
rs9317518		chr13	-3.457	0.417	0.471	0.341
rs10857111		chr4	3.452	0.520	0.362	0.008
rs9962453	FHOD3	chr18	3.435	0.534	0.390	0.008
rs9564266		chr13	-3.433	0.431	0.293	0.005
rs9957591		chr18	3.398	0.431	0.270	0.003
rs17835005		chr4	3.389	0.616	0.379	3.22E-05
rs13125280		chr4	3.389	0.616	0.379	3.22E-05
rs502154	FHOD3	chr18	3.387	0.363	0.287	0.135
rs2333484		chr14	3.361	0.417	0.557	0.016
rs2848923	FHOD3	chr18	3.358	0.5	0.344	0.005
rs7327655		chr13	3.329	0.397	0.235	0.003
rs9519707		chr13	-3.302	0.294	0.356	0.228
rs11253996	PTER	chr10	-3.289	0.520	0.425	0.105
rs12673465	CREB5	chr7	3.273	0.308	0.350	0.447
rs12360753		chr11	-3.249	0.438	0.436	0.977

rs7915486	PTER	chr10	-3.246	0.527	0.333	0.000
rs866973		chr13	3.204	0.356	0.517	0.005
rs11203367	PADI4	chr1	-3.200	0.369	0.540	0.002
rs4737859		chr8	-3.193	0.356	0.522	0.004
rs10888024	PADI4	chr1	-3.183	0.376	0.488	0.037
rs10206763	LRRTM4	chr2	-3.166	0.328	0.482	0.006
rs1362886		chr13	3.162	0.513	0.425	0.135
rs2901520	FABP6	chr5	-3.162	0.363	0.436	0.209
rs2923006		chr6	3.160	0.513	0.436	0.199
rs2156875		chr6	3.160	0.547	0.396	0.005
rs3744903	FHOD3	chr18	3.156	0.547	0.396	0.005
rs2814968		chr6	-3.108	0.356	0.287	0.162
rs11618600	DAOA-AS1	chr13	3.087	0.369	0.436	0.253
rs17238423		chr4	3.080	0.253	0.373	0.024
rs2596573		chr6	3.061	0.369	0.517	0.012
rs2596571		chr6	3.061	0.356	0.436	0.166
rs9266692		chr6	3.061	0.356	0.436	0.166
rs9266710		chr6	3.061	0.356	0.436	0.166
rs2507979		chr6	3.061	0.356	0.436	0.166
rs1523		chr6	3.061	0.356	0.436	0.166
rs4259246	UHRF1BP1	chr6	-3.058	0.513	0.431	0.184
rs1748041	PADI4	chr1	-3.058	0.363	0.281	0.108
rs11203366	PADI4	chr1	-3.058	0.239	0.166	0.109
rs2814992	C6orf106	chr6	-3.046	0.239	0.166	0.109
rs2744949	C6orf106	chr6	-3.046	0.239	0.166	0.109
rs2744943	C6orf106	chr6	-3.046	0.239	0.166	0.109
rs2814945	C6orf106	chr6	-3.046	0.239	0.166	0.109
rs4513788		chr6	-3.046	0.239	0.166	0.109
rs6838566		chr4	3.021	0.246	0.379	0.014
rs427294	LRRTM4	chr2	3.011	0.397	0.316	0.110
rs4902116		chr14	3.010	0.486	0.379	0.069
rs7219476	SLC39A11	chr17	2.999	0.541	0.425	0.037
rs10788664	PADI4	chr1	-2.989	0.438	0.298	0.008
rs6917363		chr6	2.952	0.376	0.436	0.304
rs11755393	UHRF1BP1	chr6	-2.929	0.280	0.494	0.000
rs4706273	LMBRD1	chr6	-2.900	0.280	0.494	0.000
rs1031629	LMBRD1	chr6	-2.900	0.280	0.494	0.000
rs9354880		-	-2.900	0.513	0.367	0.005
rs4799887	FHOD3	chr18	2.896	0.273	0.431	0.005
rs1390609		chr2	2.886	0.356	0.545	0.000
rs2546375		chr5	-2.886	0.342	0.488	0.008
rs28623233		chr8	-2.883	0.198	0.350	0.003
rs918476		chr5	-2.882	0.404	0.275	0.007
rs6472348		chr8	2.879	0.239	0.120	0.005
rs4621791		chr8	2.879	0.458	0.551	0.109
rs10829360		chr10	2.854	0.472	0.321	0.010
rs10125346	NFIB	chr9	-2.846	0.472	0.321	0.010
rs2303510	FHOD3	chr18	2.840	0.356	0.488	0.019
rs1081230		chr15	2.828	0.321	0.413	0.073

rs2118888	EML4	chr2	2.824	0.273	0.5	5.22E-05
rs92303		chr2	2.823	0.383	0.419	0.536
rs9586880		chr13	-2.819	0.294	0.390	0.083
rs1907333	C10orf11	chr10	-2.817	0.294	0.390	0.083
rs9571430		chr13	-2.813	0.397	0.528	0.021
rs9354887	LMBRD1	chr6	-2.808	0.630	0.436	0.000
rs2272383	TUB	chr11	-2.807	0.404	0.281	0.010
rs1748035	PADI4	chr1	-2.805	0.486	0.356	0.017
rs1748033	PADI4	chr1	-2.805	0.308	0.183	0.011
rs1031631	LMBRD1	chr6	-2.794	0.232	0.109	0.002
rs1030976	NPAS3	chr14	2.792	0.335	0.477	0.011
rs11061029	RIMBP2	chr12	2.772	0.5	0.321	0.001
rs290024	LRRTM4	chr2	2.747	0.458	0.402	0.309
rs2303509	FHOD3	chr18	2.741	0.294	0.5	0.000
rs7604971		chr2	-2.737	0.294	0.5	0.000
rs1709330	KLHL29	chr2	-2.730	0.438	0.339	0.086
rs7743613	LMBRD1	chr6	-2.729	0.321	0.448	0.020
rs7752533	LMBRD1	chr6	-2.726	0.527	0.385	0.015
rs2031446	NBEA	chr13	2.724	0.458	0.557	0.091
rs4799431	FHOD3	chr18	2.700	0.342	0.275	0.157
rs2596181	RYR3	chr15	2.697	0.328	0.5	0.001
rs2189990		chr7	-2.691	0.458	0.459	0.988
rs1976194	PRKCB	chr16	-2.685	0.561	0.425	0.013
rs9544843	NBEA	chr13	2.685	0.232	0.396	0.003
rs1246278		chr9	-2.673	0.404	0.522	0.042
rs12898670		chr15	2.657	0.513	0.333	0.001
rs644287	SCN4B	chr11	2.649	0.335	0.488	0.006
rs11633169	U6	chr15	2.638	0.335	0.488	0.006
rs1246292		chr9	-2.637	0.335	0.488	0.006
rs874881	PADI4	chr1	-2.637	0.458	0.471	0.835
rs4530905		chr6	-2.630	0.479	0.459	0.730
rs4540292		chr6	-2.630	0.404	0.431	0.649
rs4394274		chr6	-2.630	0.321	0.247	0.160
rs222468		chr2	2.627	0.410	0.327	0.122
rs759636		chr19	2.626	0.431	0.471	0.475
rs988366		chr4	2.623	0.376	0.511	0.019
rs6752455	EML4	chr2	2.619	0.383	0.293	0.102
rs6671591		chr1	-2.616	0.315	0.477	0.003
rs4758042	RIC3	chr11	-2.603	0.253	0.132	0.005
rs1018542		chr14	-2.587	0.513	0.402	0.038
rs7749324	LMBRD1	chr6	-2.582	0.438	0.310	0.019
rs10931978		chr2	-2.577	0.219	0.120	0.019
rs3809994	FHOD3	chr18	2.568	0.363	0.5	0.011
rs11645307	FA2H	chr16	2.559	0.226	0.425	0.000
rs4453730		chr2	-2.552	0.301	0.402	0.049
rs8035021	RYR3	chr15	-2.552	0.417	0.229	0.000
rs1538814		chr13	-2.550	0.452	0.339	0.023
rs4426407	SLC39A11	chr17	2.550	0.301	0.517	5.75E-05
rs1919951		chr7	2.547	0.321	0.270	0.355

rs12133002	RYR2	chr1	2.536	0.184	0.333	0.003
rs11725593		chr4	2.535	0.198	0.327	0.011
SNP1-113796951		-	2.531	0.397	0.264	0.010
rs479245		chr4	2.522	0.390	0.235	0.003
rs7552954	MAGI3	chr1	2.521	0.561	0.390	0.003
rs1936930	MAGI3	chr1	2.521	0.328	0.270	0.254
SNP1-113785445		-	2.521	0.342	0.408	0.237
rs10776770	MAGI3	chr1	2.521	0.404	0.557	0.008
SNP1-113792062		-	2.521	0.513	0.402	0.049
rs10157946	MAGI3	chr1	2.521	0.397	0.557	0.005
SNP1-113855400		-	2.521	0.397	0.557	0.005
SNP1-113865115		-	2.521	0.397	0.557	0.005
rs1548111		chr4	2.518	0.397	0.557	0.005
rs4969121	SLC39A11	chr17	2.514	0.397	0.557	0.005
rs9266716		chr6	2.514	0.397	0.557	0.005
rs11807523		chr1	-2.507	0.397	0.557	0.005
rs11761517	ESYT2	chr7	-2.501	0.397	0.557	0.005
rs10193935	EML4	chr2	2.498	0.410	0.235	0.000
rs1320893		chr7	2.495	0.253	0.465	0.000
rs7866589	NFIB	chr9	-2.495	0.424	0.344	0.115
rs282802		chr15	-2.492	0.390	0.258	0.012
rs10195776	EML4	chr2	2.492	0.280	0.201	0.090
rs4758309		chr11	-2.474	0.212	0.120	0.029
rs1514033	RYR3	chr15	-2.457	0.410	0.241	0.001
rs4809501		chr20	-2.455	0.205	0.120	0.043
rs6856727		chr4	2.449	0.321	0.465	0.005
rs7327540	NBEA	chr13	2.449	0.328	0.178	0.002
rs7985429	NBEA	chr13	2.435	0.438	0.477	0.509
rs349235		chr4	2.429	0.232	0.333	0.065
rs1360507	LINGO2	chr9	-2.410	0.301	0.431	0.015
rs6973629		chr7	2.409	0.445	0.477	0.589
rs7209417	SEPT9	chr17	2.400	0.5	0.373	0.026
rs1983248		chr1	2.391	0.205	0.333	0.010
rs13210323	ANKS1A	chr6	-2.387	0.315	0.281	0.522
rs11870986	BAIAP2	chr17	-2.380	0.410	0.563	0.005
rs1838041		chr8	2.373	0.301	0.201	0.032
rs12104046		chr18	2.368	0.417	0.517	0.074
rs6048760	GZF1	chr20	2.366	0.527	0.471	0.340
rs4937911		chr11	-2.358	0.534	0.442	0.105
rs6930785		chr6	-2.348	0.397	0.528	0.018
rs10954922		chr8	2.347	0.287	0.390	0.050
rs10857712	MTG1	chr10	2.347	0.465	0.344	0.028
rs7807163		chr7	-2.338	0.417	0.287	0.024
rs2041632	ANLN	chr7	2.333	0.547	0.436	0.047
rs10753623		chr1	-2.333	0.493	0.511	0.750
rs4357857		chr14	2.329	0.541	0.471	0.229
rs2222025		chr18	-2.326	0.390	0.563	0.002
rs11041793		chr11	2.326	0.424	0.402	0.704
rs1464108	RIMBP2	chr12	-2.326	0.431	0.281	0.009

rs17702687	CFL1P1	chr10	-2.321	0.280	0.189	0.078
rs2961144	OR2A5	chr7	2.318	0.452	0.505	0.341
rs10790230	TMPRSS4	chr11	2.310	0.582	0.448	0.013
rs10171643	EML4	chr2	2.302	0.513	0.431	0.135
rs3845534		chr1	-2.301	0.534	0.482	0.367
rs1029937	GABRG3	chr15	2.295	0.424	0.574	0.009
rs7819605		chr8	-2.293	0.383	0.235	0.004
rs9822586	EIF4E3	chr3	-2.291	0.376	0.551	0.001
rs7033700	TTLL11	chr9	2.288	0.226	0.270	0.375
rs2769332	NBEA	chr13	2.278	0.431	0.344	0.115
rs4636658		chr11	-2.273	0.328	0.522	0.000
rs13375	EML4	chr2	2.271	0.198	0.109	0.030
rs6843010	GALNTL6	chr4	-2.263	0.417	0.408	0.870
rs7299202		chr12	2.262	0.356	0.333	0.700
rs4794135		chr17	2.256	0.356	0.333	0.700
rs11079913	XYLT2	chr17	2.256	0.308	0.270	0.461
rs1566851		chr10	-2.254	0.356	0.316	0.439
rs139998	TXN2	chr22	-2.251	0.431	0.362	0.150
rs7044278		chr9	2.251	0.410	0.534	0.028
rs9531151		chr13	2.250	0.410	0.534	0.028
rs1995622		chr11	2.249	0.465	0.528	0.252
rs2294057	MYOM2	chr8	-2.242	0.486	0.373	0.040
rs2044607	EIF4E3	chr3	-2.240	0.404	0.448	0.439
rs847851	ANKS1A	chr6	-2.239	0.458	0.465	0.912
rs3215537	TAF11	chr6	-2.239	0.102	0.195	0.036
rs4571496		chr5	-2.238	0.383	0.511	0.037
rs6062830		chr20	-2.233	0.239	0.132	0.014
rs651474	TMPRSS4	chr11	2.231	0.116	0.201	0.030
rs2024031		chr7	-2.230	0.232	0.339	0.030
rs718939		chr7	-2.230	0.493	0.379	0.041
rs1943539		chr18	2.229	0.226	0.212	0.779
rs13333251		chr16	-2.228	0.226	0.212	0.779
rs4793651	MRPL27	chr17	2.227	0.328	0.189	0.004
rs6817219		chr4	2.225	0.246	0.362	0.025
rs11976037		chr7	2.224	0.479	0.454	0.668
rs6477714		chr9	-2.224	0.589	0.425	0.006
SNP4-9767609		-	2.218	0.438	0.540	0.080
SNP4-9768555		-	2.218	0.328	0.465	0.016
rs12090228		chr1	-2.214	0.267	0.367	0.053
rs6799805		chr3	-2.213	0.191	0.362	0.000
rs6016431		chr20	2.205	0.458	0.385	0.178
rs1881229		chr11	-2.203	0.417	0.471	0.335
rs11041788		chr11	-2.202	0.458	0.339	0.025
rs9827586	MME	chr3	2.198	0.232	0.201	0.514
rs10952737	CNTNAP2	chr7	2.198	0.397	0.568	0.003
rs1460346		chr4	2.196	0.513	0.385	0.023
rs7855642		chr9	2.192	0.356	0.235	0.015
rs207954	SLCO3A1	chr15	-2.189	0.232	0.143	0.051
rs1870576		chr15	-2.185	0.232	0.143	0.051

rs11649073		chr16	-2.184	0.376	0.471	0.084
rs5021051	GRID2	chr4	2.183	0.410	0.505	0.086
rs9370524		chr6	-2.179	0.452	0.339	0.028
rs1246264		chr9	-2.176	0.280	0.316	0.517
rs1432444	LIPF	chr10	-2.170	0.246	0.356	0.034
rs905571	GALNTL6	chr4	-2.165	0.321	0.471	0.005
rs12266194		chr10	2.157	0.390	0.488	0.066
rs986980	NRXN3	chr14	2.157	0.178	0.074	0.002
rs926014		chr2	2.157	0.047	0.189	0.000
rs43870		chr8	-2.156	0.047	0.189	0.000
rs3911309		chr11	-2.155	0.445	0.333	0.033
rs6010873		chr20	-2.155	0.404	0.327	0.169
rs225359	TFF1	chr21	-2.151	0.383	0.264	0.037
rs1289004		chr1	2.151	0.589	0.413	0.003
rs2638148		chr3	2.150	0.301	0.413	0.034
rs6969989	CD36	chr7	-2.149	0.328	0.183	0.002
rs440238	PTPRD	chr9	-2.147	0.143	0.316	0.000
rs1391228		chr16	-2.146	0.349	0.557	0.000
rs13226728	OR2A5	chr7	2.145	0.356	0.5	0.015
rs931350	LRRC4C	chr11	2.144	0.212	0.287	0.124
SNP1-114044395		-	-2.140	0.287	0.178	0.021
SNP1-114057351		-	-2.140	0.417	0.477	0.307
rs6062422		chr20	-2.138	0.047	0.195	0.000
rs13148469	ODZ3	chr4	-2.131	0.047	0.195	0.000
rs2738169		chr8	-2.131	0.205	0.379	0.000
rs7866986		chr9	-2.130	0.205	0.379	0.000
SNP4-9754831		-	2.130	0.205	0.379	0.000
rs10763263		chr10	-2.125	0.178	0.304	0.009
rs1333015		chr13	-2.124	0.328	0.396	0.170
rs1061820	NFIB	chr9	-2.121	0.404	0.373	0.610
SNP1-114081658		-	-2.117	0.047	0.183	0.000
SNP1-114090214		-	-2.117	0.383	0.436	0.366
rs8667	ATF5	chr19	-2.117	0.301	0.178	0.012
rs3170545	ATF5	chr19	-2.117	0.308	0.511	0.000
rs8647	ATF5	chr19	-2.117	0.390	0.258	0.011
rs540259	PTPRD	chr9	2.115	0.294	0.459	0.001
rs4245047		chr11	-2.114	0.287	0.425	0.020
rs2024746	NAPB	chr20	2.113	0.404	0.522	0.042
rs2253834	NAPB	chr20	2.112	0.493	0.471	0.711
rs11976180		chr7	2.110	0.383	0.241	0.005
rs358706	PLCH1	chr3	-2.109	0.376	0.201	0.001
rs1440997	PTPRM	chr18	-2.105	0.328	0.183	0.005
SNP1-114044229		-	-2.104	0.328	0.183	0.005
rs778291		chr13	-2.101	0.376	0.557	0.001
rs6857474		chr4	2.100	0.232	0.137	0.034
rs487740		chr5	2.098	0.376	0.540	0.004
rs613105	PTPRD	chr9	-2.095	0.047	0.183	0.000
rs1809134		chr2	-2.091	0.047	0.183	0.000
rs11130703		chr3	2.090	0.301	0.459	0.003

rs247362		chr12	-2.081	0.226	0.327	0.048
rs247361		chr12	-2.081	0.410	0.563	0.007
rs3894571	CRELD1	chr3	-2.070	0.582	0.385	0.000
rs1220789		chr9	2.068	0.363	0.540	0.002
rs6741441		chr2	2.065	0.479	0.505	0.649
rs10761942		chr10	-2.064	0.479	0.505	0.649
SNP1-114086826		-	-2.058	0.363	0.235	0.010
SNP1-114094411		-	-2.058	0.363	0.580	6.37E-05
SNP16-73543017		-	2.058	0.342	0.160	0.000
rs658624	SCN4B	chr11	-2.057	0.582	0.379	0.000
rs2275287	RYR2	chr1	-2.055	0.356	0.551	0.000
rs6729116		chr2	-2.055	0.301	0.195	0.021
rs1327068		chr1	-2.052	0.260	0.350	0.076
SNP4-9787333		-	2.043	0.328	0.454	0.020
rs239622		chr21	-2.041	0.191	0.333	0.002
rs11000440	OIT3	chr10	2.038	0.390	0.402	0.829
rs6552102	CENPC1	chr4	2.035	0.404	0.511	0.057
rs1464023		chr2	2.034	0.315	0.160	0.002
rs9787820	LRRC4C	chr11	-2.031	0.513	0.436	0.168
rs1512454		chr6	2.031	0.205	0.373	0.000
rs10107668		chr8	2.028	0.404	0.551	0.008
rs11736516		chr4	2.025	0.417	0.505	0.141
rs13397241		chr2	2.022	0.164	0.063	0.003
rs6973242	CD36	chr7	-2.021	0.308	0.459	0.006
rs13026918	MRPL19	chr2	2.019	0.226	0.040	7.81E-06
rs9266042		chr6	2.019	0.246	0.350	0.041
rs226822		chr20	-2.015	0.301	0.206	0.060
rs2596548		chr6	2.014	0.349	0.224	0.008
rs2596549		chr6	2.014	0.513	0.390	0.023
rs2596427		chr6	2.014	0.438	0.482	0.423
rs2254556		chr6	2.014	0.493	0.321	0.003
rs4959017		chr6	2.014	0.280	0.298	0.723
rs3997982		chr6	2.014	0.212	0.120	0.019
rs9266596		chr6	2.014	0.554	0.471	0.150
rs4959070		chr6	2.014	0.273	0.413	0.009
